# Mathematical and experimental analysis of localization of anti-tumour antibody–enzyme conjugates

**DOI:** 10.1038/sj.bjc.6690592

**Published:** 1999-08

**Authors:** T L Jackson, S R Lubkin, N O Siemers, D E Kerr, P D Senter, J D Murray

**Affiliations:** Department of Applied Mathematics, University of Washington, Box 352420, Seattle, WA 98195-2420, USA; Bristol-Myers Squibb Pharmaceutical Research Institute, 3005 First Avenue, Seattle, WA 98121, USA

## Abstract

Considerable research has been aimed at improving the efficacy of chemotherapeutic agents for cancer therapy. A promising two-step approach that is designed to minimize systemic drug toxicity while maximizing activity in tumours employs monoclonal antibody (mAb)–enzyme conjugates for the activation of anticancer prodrugs. We present, analyse and numerically simulate a mathematical model based on the biology of the system to study the biodistribution, pharmacokinetics and localization properties of mAb–enzyme conjugates in tumour tissue. The model predictions were compared with experimental observations and an excellent correlation was found to exist. In addition, the critical parameters affecting conjugate half-life were determined to be the inter-capillary half-distance and the antibody–antigen binding affinity. An approximation is presented relating the per cent injected dose per gram to inter-capillary half-distance and time. Finally, the model was used to examine various dosing strategies in an attempt to determine which regimen would provide the best biodistribution results. We compared the results of administering a uniform dose of fusion protein via bolus injection, multiple injections and continuous infusion. The model predicts that dosing strategy has little effect on the amount of conjugate that localizes in the tumour. © 1999 Cancer Research Campaign


*British Journal of Cancer* (1999) **80**(11), 1747–1753
© 1999 Cancer Research Campaign
Article no. bjoc.1998.0592

### Mathematical and experimental analysis of localization of anti-tumour antibody–enzyme conjugates

**TL Jackson[1], SR Lubkin[1], NO Siemers[2], DE Kerr[2], PD Senter[2] and JD Murray[1]**

[1]Department of Applied Mathematics, University of Washington, Box 352420, Seattle WA 98195-2420, USA; [2]Bristol-Myers Squibb Pharmaceutical Research Institute, 3005 First Avenue, Seattle, WA 98121, USA

**Summary** Considerable research has been aimed at improving the efficacy of chemotherapeutic agents for cancer therapy. A promising two-step approach that is designed to minimize systemic drug toxicity while maximizing activity in tumours employs monoclonal antibody (mAb)–enzyme conjugates for the activation of anticancer prodrugs. We present, analyse and numerically simulate a mathematical model based on the biology of the system to study the biodistribution, pharmacokinetics and localization properties of mAb–enzyme conjugates in tumour tissue. The model predictions were compared with experimental observations and an excellent correlation was found to exist. In addition, the critical parameters affecting conjugate half-life were determined to be the inter-capillary half-distance and the antibody–antigen binding affinity. An approximation is presented relating the per cent injected dose per gram to inter-capillary half-distance and time. Finally, the model was used to examine various dosing strategies in an attempt to determine which regimen would provide the best biodistribution results. We compared the results of administering a uniform dose of fusion protein via bolus injection, multiple injections and continuous infusion. The model predicts that dosing strategy has little effect on the amount of conjugate that localizes in the tumour.

Much research has surrounded the use of monoclonal antibody (mAb)–enzyme conjugates for the activation of anticancer prodrugs (Melton and Sherwood, 1996; Niculescu-Duvaz and Springer, 1996; Senter and Svensson, 1997). This is a two-step approach to drug delivery in which a mAb–enzyme conjugate that specifically localizes into solid tumour masses is administered, followed by systemic treatment with an anticancer prodrug. Upon contact with the targeted enzyme, the prodrug is converted into an active cytotoxic drug. The advantages of this targeting strategy over the use of covalently linked mAb–drug conjugates (Pietersz et al, 1994) for selective drug delivery are that a single localized mAb–enzyme conjugate is capable of catalytically generating large amounts of active drug, and the drug thus formed can penetrate into regions of the tumour mass that are inaccessible to the conjugate. Pharmacokinetics studies have demonstrated that mAb–enzyme/prodrug combinations can result in high intratumoural drug concentrations (Bosslet et al, 1994; Wallace et al, 1994; Svensson et al, 1995), and pronounced anti-tumour activities in an array of preclinical tumour models (Springer et al, 1991; Meyer et al, 1993; Eccles et al, 1994; Kerr et al, 1995; Siemers et al, 1997) and in a small scale clinical trial have been reported (Bagshawe and Begent, 1996).

A number of critical issues must be addressed to assure optimal therapeutic efficacy with this targeting strategy. In order to minimize systemic non-targeted drug release, a high mAb–enzyme tumour to normal tissue ratio is needed before the prodrug is administered. The time required for this to occur varies among the many mAb-enzyme/prodrug systems reported. For example, in nude mouse models for human cancer, the delay between conjugate and prodrug administration was 12 h for the recombinant L49-sFv-bL fusion protein (molecular mass 66.5 kDa) (Siemers et al, 1997), 3 days for 96.5-Fab´-bL (molecular mass 92 kDa) (Kerr et al, 1995), 1 week for the anti-CEA-Fab-$\beta$-glucuronidase fusion protein (molecular mass 250 kDa) (Bosslet et al, 1994) and 2 weeks for the ICR12-carboxypeptidase G2 (molecular mass 233–316 kDa) (Eccles et al, 1994). Further critical parameters include the absolute amount of conjugate that localizes within the tumour mass, the enzyme kinetic parameters ($K_m$ and $k_{cat}$ values) for the prodrug substrate, and the amount and potency of the drug that is released intratumourally.

Recognizing the degree of complexity inherent in this approach, Jain and co-workers have developed a model designed to predict the effects of conjugate distribution and enzyme kinetic parameters on intratumoural and systemic drug concentrations (Baxter et al, 1992; Baxter and Jain, 1996). This model provides a theoretical underpinning for the need to have high tumour to blood conjugate ratios before prodrug is administered. Since residual mAb–enzyme conjugate in the blood will lead to systemic drug release, the model of Baxter and Jain leads to the prediction that for a particular prodrug, activating enzymes with relatively higher $K_m$ and lower $k_{cat}$ values would be expected to provide higher intratumoural drug concentrations.

Based on the fact that conjugate tumour localization and retention is critical for the therapeutic efficacy in many mAb-based targeting strategies including the one described here, we wished to determine which of the many possible critical issues are most influential. This work describes a mathematical model that analyses the effects that many of the physiological and biochemical parameters have on intratumoural uptake of a mAb–enzyme conjugate. The model suggests that vascularity and the kinetics of conjugate dissociation from tumour antigens are critical determinants for intratumoural conjugate localization and retention. Experiments are presented that provide validation for this mathematical model.

*Received 3 April 1998*
*Revised 29 September 1998*
*Accepted 4 October 1998*

*Correspondence to:* JD Murray

### MATERIALS AND METHODS

### Expression of L49-sFv-bL

L49-sFv-bL, which is comprised of the single chain Fv fragment of the L49 antibody fused to a mutated form of *Escherichia cloacae* bL, was expressed in *E. coli* and purified from culture supernatants as previously described (Siemers et al, 1997). Briefly, the *E. coli* strain BL21(λDE3) was transformed with the plasmid encoding L49-sFv-bL fused to the pelB leader sequence. The gene orientation was pelB-$V_H$-218 linker-$V_L$-bL. Soluble expression of L49-sFv-bL was accomplished at 23°C with 0.05 mM isopropyl β-D-thiogalactopyranoside to induce protein expression. The fusion protein was purified from the culture supernatant using a two-step affinity purification technique involving binding of the L49 portion of the protein to immobilized p97 antigen, followed by binding of the bL portion to immobilized phenylboronic acid. The protein obtained in this manner was pure by sodium dodecyl sulphate polyacrylamide gel electrophoresis (SDS-PAGE) and retained the the activities of both the L49 and the bL moieties.

Surface plasmon resonance experiments were performed on a BIAcore 2000 instrument (Pharmacia) as previously described (Siemers et al, 1997). The results from four independent experiments led to association and dissociation, $k_a$ and $k_d$ respectively, values of $3.88 \times 10^5$ $M^{-1}$ $s^{-1}$ and $4.83 \times 10^{-4}$ $s^{-1}$ respectively.

### Conjugate localization

The 3677 melanoma tumour line (Kerr et al, 1995) was implanted subcutaneously into the flanks of female athymic *nu/nu* mice (8–12 weeks old, Harlan Sprague-Dawley, Indianapolis, IN, USA) and were allowed to grow for 8–10 days, at which time the tumours were approximately 150 $mm^3$ in volume. The mice were injected with L49-sFv-bL (1.33 mg $kg^{-1}$ $injection^{-1}$ at 4-h intervals for three doses, or 4 mg $kg^{-1}$ in a single dose), and at various time intervals, the mice were anaesthetized, bled through the orbital plexus and sacrificed. The tumours were removed, weighed and homogenized in phosphate-buffered saline (PBS), pH 7.2, containing 15 μg $ml^{-1}$ aprotinin (2 ml $g^{-1}$ of tissue). To the homogenate was added 50 mM sodium phosphate containing 100 mM sodium chloride at pH 11.2 (10 ml $g^{-1}$ of tissue), and the suspension was mixed. After 20 min at room temperature, 3 M sodium phosphate at pH 7.0 were added (2 ml $g^{-1}$ of tissue), and the mixture was mixed and centrifuged.

Quantification of conjugate concentration was accomplished by determining the bL activity in the tumours of treated mice compared to untreated control tumours, and tumours that were spiked with known quantities of L49-sFv-bL just prior to extraction. Polystyrene 96-well microtitre plates were coated with an affinity-purified rabbit polyclonal antiserum to wild-type *E. cloacae* bL (1 μg $ml^{-1}$, and were then blocked with specimen diluent (Genetic Systems Corp., Seattle, WA, USA). Serially diluted tissue extracts or purified samples were added to the wells and allowed to bind for 3 h at room temperature. The plates were washed with specimen diluent and were developed by adding 0.1 ml of nitrocefin (O'Callaghan et al, 1972) at 0.1 mM in PBS, pH 7.2, containing 1% dimethylformamide. Absorbance measurements were read in an enzyme-linked immunosorbent assay (ELISA) plate reader using a 490 nm filter with 630 nm as the reference wavelength.

### Model

The mathematical model is based on the conception of the tumour as equivalent to a collection of cylinders of tissue, each nourished by a single capillary, the collection possessing the same structure as a box of pencils (Krogh, 1919). Each Krogh cylinder is considered to have an outer radius $L$ equal to half the average distance between capillary centres and an inner radius $\rho$ equal to the average radius of the tumour capillaries.

The pharmacokinetics outside the tumour is modelled by two well-mixed compartments, the plasma compartment and the peripheral compartment representing all other non-tumour, non-plasma tissues. The fusion protein is injected directly into the plasma compartment and distributes throughout via reversible transfer between the plasma and peripheral compartments. The fusion protein can only be eliminated from the system while in the plasma compartment and only from here can it pass through the microvessel wall and enter the tissue. The tissue is considered to be homogeneous, that is the intracellular and extracellular space is considered to be one unit. Once in the tissue compartment the fusion protein is free to diffuse or to bind to antigens presented on cell surfaces. Since the fusion protein has an isoelectric point near neutrality (Siemers et al, 1997), we have not factored in the effect of protein net charge on biodistribution and clearance.

The usual compartment approach yields the mathematical model of Baxter and Jain (1996) which describes the rate of change of the concentration of free and bound fusion protein in the tumour, plasma and peripheral compartments. The variables that are tracked are listed in Table 1 along with the parameters that govern their rates of change.

In the microvessel, the concentration of conjugate, $p_1$, satisfies

$$\frac{dp_1}{dt} = -\ k_{12}p_1 \ +\ k_{21}\,p_2 \ -\ k_e p_1 \ +\ I(t)$$

Rate of change of fusion protein = Transfer Out + Transfer In – Elimination + Dosing

In the peripheral compartment, the concentration of conjugate, $p_2$, satisfies

$$\frac{dp_2}{dt} = \ k_{12}p_1 \ -\ k_{21}\,p_2$$

Rate of change of fusion protein = Transfer In – Transfer Out

In the tissue, the concentrations of free conjugate, $F_F$, and bound conjugate, $F_B$, are governed by

$$\frac{\partial F_F}{\partial t} = \ D_F \Delta F_F \ -\ k_a F_F (A_0 - F_B) \ +\ k_d F_B$$

Rate of change of free fusion protein = Diffusion – Binding + Dissociation

$$\frac{\partial F_B}{\partial t} = \ k_a F_F (A_0 - F_B) \ -\ k_d F_B$$

Rate of change of bound fusion protein = Binding – Dissociation where Δ is the cylindrically symmetric Laplacian operator in three dimensions ($\Delta = \frac{\partial^2}{\partial r^2} + \frac{1}{r}\frac{\partial}{\partial r}$).

The initial concentration of fusion protein in each compartment must be given and boundary conditions governing the behaviour at the boundaries must be prescribed to complete the model. Initially, the concentration of both free and bound fusion protein in the tumour is zero:

 *© 1999 Cancer Research Campaign*

$F_F(r, 0) = 0$
$F_B(r, 0) = 0$

The initial concentration of fusion protein in the plasma compartment is the administered dose, $F_0$, and there is no fusion protein in the peripheral compartment initially:

$p_1(0) = F_0$
$p_2(0) = 0$

Due to symmetry with surrounding Krogh cylinders, the net flux across the tissue boundary, $r = L$, is zero:

$$\frac{\partial F_F}{\partial r}(L,t) = 0$$

The flux across the blood vessel wall, $r = \rho$, is driven by the concentration difference in the two compartments and by the wall permeability.

$$\frac{\partial F_F}{\partial r}(\rho,t) = -\frac{P}{D_F}[F_1(t) - F_F(p,t)]$$

The mathematical model equations were solved by computer simulation, using a finite difference scheme and different experimental scenarios were tested.

### Data analysis

By incorporation of the experimental data, a least-squares fit for the plasma clearance of the fusion protein, L49-sFv-bL (Siemers et al, 1997), into the biexponential function $C = Ae^{-\alpha t} + Be^{-\beta t}$ was obtained and values for $A$, $B$, $\alpha$, $\beta$ were determined. These values were in turn used to compute the compartmental transfer coefficients $k_{12}$, $k_{21}$ and $k_e$. The diffusion coefficient, $D_F$, was assumed to depend on the molecular weight, $M_W$ as $a(M_W)^b$ using published values for $a$ and $b$ (Nugent and Jain, 1984). To compute the antigen density, $A_0$, the average value of the number of molecules per cell when saturated was $2.1 \times 10^4 \frac{\text{molecules}}{\text{cell}}$. The cell density was estimated as $2.83 \times 10^{11} \frac{\text{cell}}{\text{litre}}$ (Baxter and Jain, 1991) by assuming 50% cell fraction and an average cancer cell diameter of 15 $\mu$m (cell density = $\frac{\text{cell fraction}}{\left(\frac{4}{3}\right)\pi r^3}$). Together these values lead to the antigen density listed in Table 2. The average distance between capillary centres was calculated as $L = \sqrt{\frac{0.25}{\pi N}}$ where $N$ is the average number of capillaries in every 0.25 mm² of tissue. $N$ was determined experimentally on parafirmaldehyde fixed tumours that were embedded in paraffin.

### RESULTS

We calculated the per cent injected dose of conjugate per gram of tumour tissue as the average in a complete Krogh cylinder at a given time post-injection of conjugate. Model parameters were varied to determine which were most influential for the localization and retention of conjugate within the tumour mass.

### Dependence on tumour vasculature

In a poorly vascularized section of the tumour, the Krogh cylinders are large and each microvessel must supply more of the surrounding tissue. As a result the per cent injected dose per gram observed in the tumour at a given time after injection decreases rapidly with Krogh cylinder size (Figure 1A). The dependence of the per cent injected dose at a given time post-injection, on the Krogh cylinder size or inter-capillary half-distance, $L$, was found to follow a power law. There is approximately a straight-line fit (with slope $m < 0$) of the log–log plot of per cent injected dose vs. $L$ in the experimentally determined range of $L$ (shaded regions, Figure 1B). Thus we can write

$$\% \text{ Injected dose} = m_0 L^{m(t)}$$

where $m_1(t)$ is the time-dependent slope of the line in Figure 1B, which was found to vary linearly with time. That is $m(t) = m_1 + m_2 t$. This leads to an exponential decrease in the per cent injected dose over time, and we can write

$$\% \text{ Injected dose} = Pe^{-\mu t}$$

where $P = P(L) = m_0 L^{m_1}$ and $\mu = \mu(L) = -m_2\log(L)$. Given the parameter values in Table 2, we find $m_1 = -2.2$, $m_2 = 0.037$, and $m_0 = 0.015$. Figure 2 compares this asymptotic approximation (Jackson et al, manuscript in preparation) to the numerical solution.

**Table 1** Definition of variables and parameters of the model

| Variable | Definition |
|---|---|
| $F_F(r,t)$ | Concentration of free fusion protein in the tissue (M) |
| $F_B(r,t)$ | Concentration of bound fusion protein in the tissue (M) |
| $p_1(t)$ | Concentration of fusion protein in the plasma (M) |
| $p_2(t)$ | Concentration of fusion protein in the peripheral compartment (M) |
| Parameter | Definition |
| $F_0$ | Initial concentration of fusion protein (M) |
| $k_{12}$, $k_{21}$ | Compartmental transfer coefficients ($s^{-1}$) |
| $k_e$ | Plasma elimination rate ($s^{-1}$) |
| $L$ | Krogh cylinder radius (mm) |
| $\rho$ | Microvessel radius (mm) |
| $P$ | Permeability coefficient of the microvessel wall (mm $s^{-1}$) |
| $D_F$ | Diffusion coefficient of fusion protein in tissue ($mm^2$ $s^{-1}$) |
| $k_a$, $k_d$ | Association and dissociation rate ($M^{-1}$ $s^{-1}$, $s^{-1}$) |
| $A_0$ | Antigen density (M) |

### Critical parameters

We investigated the effect of each parameter of the model on the elimination rate, $\mu$, of fusion protein in the tumour by calculating $E = \frac{\mu_2 - \mu_{1/2}}{\mu_1}$. Here, $\mu_1$ is the elimination rate when all parameters have the values listed in Table 2 and $\mu_{1/2}$ and $\mu_2$ are the elimination rates associated with reducing by half and doubling each parameter respectively (Table 3). The baseline $\mu_1$ for parameters in Table 2 was 0.076 $h^{-1}$ representing a half-life of 9.12 h. The parameters that have the greatest effect on the conjugate retention in the tumour are the inter-capillary half-distance, $L$, the antigen association/dissociation rates, $k_a$, $k_d$, the initial concentration of binding sites $A_0$, and the permeability, $P$.

If $L$ is small corresponding to a well vascularized section of the tumour, the half-life is significantly shorter than when $L$ is large corresponding to a poorly vascularized section of tissue. Deceasing the dissociation ratio, $\frac{k_d}{k_a A_0}$, will also significantly decrease the tumour elimination rate; and decreasing the permeability ratio, $\frac{P}{L}$, will have similar effects.

### Correlation between the mathematical model and the experimental data

The biodistribution of L49-sFv-bL in nude mice bearing subcutaneous 3677 melanoma xenografts has previously been reported

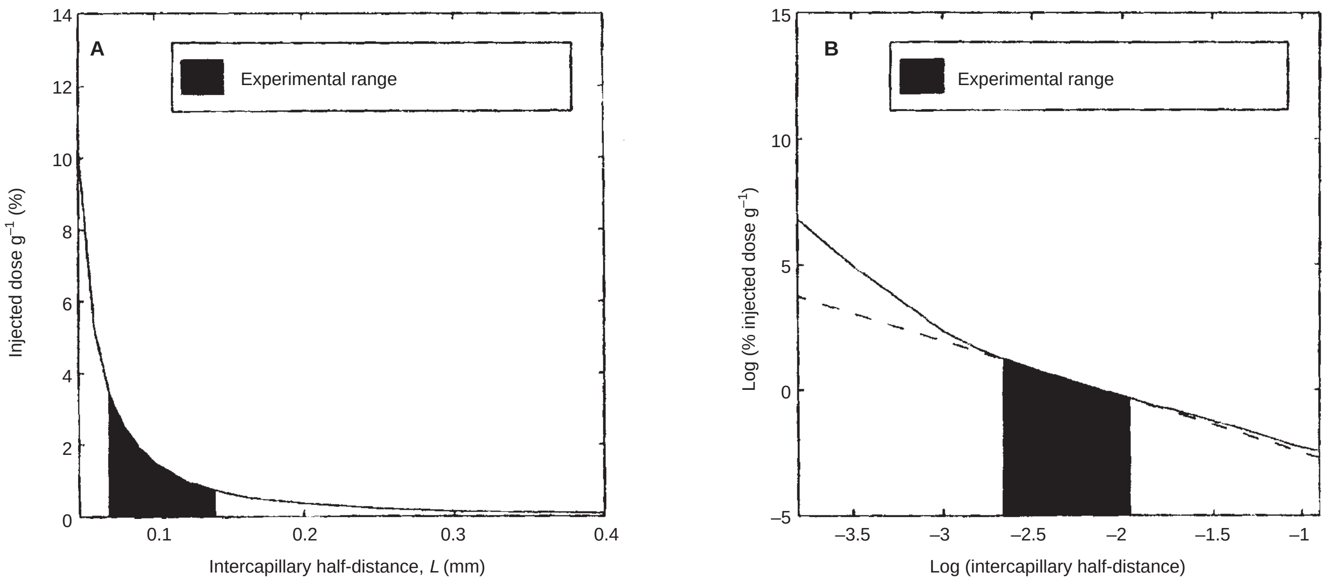

**Figure 1** Percent injected dose of fusion protein per gram of tumour tissue at $t$ = 4 h vs Krogh cylinder size (**A**), (**B**) and a log–log plot of the same information. Shaded regions in (**A**) and (**B**) correspond to the range of inter-capillary half-distances, $L$, as experimentally determined for the 3677 line

**Table 2** List of baseline parameter values used in simulations

| Parameter | Value | Reference |
|---|---|---|
| $L$ | 0.11 mm | Materials and Methods |
| $\rho$ | 0.002 mm | Materials and Methods |
| $F_0$ | $1.2 \times 10^{-7}$ M | (Siemers et al, 1997)[a] |
| $k_{12}$ | $2.4 \times 10^{-5}$ s$^{-1}$ | (Siemers et al, 1997)[b] |
| $k_{21}$ | $5.8 \times 10^{-4}$ s$^{-1}$ | (Siemers et al, 1997)[b] |
| $k_e$ | $7.1 \times 10^{-5}$ s$^{-1}$ | (Siemers et al, 1997)[b] |
| $D_F$ | $8.0 \times 10^{-6}$ mm$^2$s$^{-1}$ | (Baxter and Jain 1991) |
| $k_a$ | $3.88 \times 10^{5}$ M$^{-1}$ s$^{-1}$ | Materials and Methods |
| $k_d$ | $4.83 \times 10^{-4}$ s$^{-1}$ | Materials and Methods |
| $P$ | $9.0 \times 10^{-5}$ mm s$^{-1}$ | (Baxter and Jain, 1996) |
| $A_0$ | $1.0 \times 10^{-8}$ M | (Baxter and Jain 1991; Siemers et al, 1997) |

[a] Based on an injection of 1 mg kg$^{-1}$ conjugate ($M_w$ = 63 kDa) and a blood volume of 2.5 ml. [b] A least-squares fit for the plasma clearance of L49-sFv-bL (Siemers et al, 1997), was used to compute the compartmental transfer coefficients and the plasma elimination rate, $k_{12}$, $k_{21}$ and $k_e$.

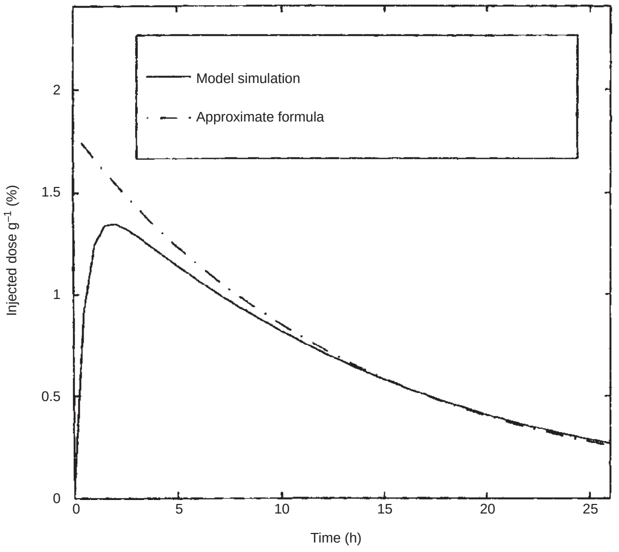

**Figure 2** Per cent injected dose of fusion protein per gram of tumour tissue vs time as predicted by the mathematical model (solid line) and the asymptotic approximation to this solution (dashed line)

(Siemers et al, 1997). In these studies, L49-sFv-bL was administered as a single bolus intravenous injection at doses of either 1 mg kg$^{-1}$ or 4 mg kg$^{-1}$. At various time points, tumours and blood were removed, and bL activity was used to assess conjugate concentration. The data from the experiments in which animals received 1 mg kg$^{-1}$ L49-sFv-bL are shown in Figure 3. This Figure also shows the results of the mathematical model simulations using the parameters listed in Table 2.

An excellent correlation exists between the model and the experimental data, both for the L49-sFv-bL concentrations in the tumour (Figure 3A) and in the plasma (Figure 3B). Table 4 shows this correlation.

### Effect of injection schedule on fusion protein localization

In order to determine the best dosing strategy for fusion protein localization, the results of administering a uniform dose of fusion protein via bolus injection, multiple injections and continuous infusion were simulated. For the bolus strategy, the injection of a single dose of $4\,\frac{mg}{kg}$ was numerically simulated and the level of fusion protein in the tumour and plasma were calculated 24 h later. For the multiple injection strategy, the administration of three equal doses of $1.33\,\frac{mg}{kg}$ at 4-h intervals was simulated so that the total amount injected was $4\,\frac{mg}{kg}$. Twenty-four hours after the initial injection, the levels of fusion protein in the tumour and the plasma were calculated. Finally, continuous infusion of $4\,\frac{mg}{kg}$ over an 8-h period was simulated, again computing the tumour and plasma levels 16 h after the end of dosing. The per cent injected dose of conjugate per gram of tumour for all three strategies as determined

*© 1999 Cancer Research Campaign*

**Table 3** The effect of each parameter on the elimination rate, $\mu$, in the tumour

| Parameter | Value | $\mu_{1/2}$ ($h^{-1}$)[a] | $\mu_2$ ($h^{-1}$)[a] | E[b] |
|---|---|---|---|---|
| $D$ | $8.0 \times 10^{-6}$ mm² s⁻¹ | 0.075 | 0.077 | 0.031 |
| $k_a$ | $3.88 \times 10^{5}$ M⁻¹ s⁻¹ | 0.14 | 0.04 | –1.3 |
| $k_d$ | $4.83 \times 10^{-4}$ s⁻¹ | 0.040 | 0.14 | 1.3 |
| $A_0$ | $1.0 \times 10^{-8}$ M | 0.14 | 0.04 | –1.3 |
| $k_{12}$ | $2.4 \times 10^{-5}$ s⁻¹ | 0.077 | 0.074 | –0.034 |
| $k_{21}$ | $5.8 \times 10^{-4}$ s⁻¹ | 0.072 | 0.078 | 0.068 |
| $k_e$ | $7.1 \times 10^{-5}$ s⁻¹ | 0.076 | 0.075 | –0.010 |
| $P$ | $9.0 \times 10^{-6}$ mm s⁻¹ | 0.04 | 0.15 | 1.5 |
| $L$ | 0.10 mm | 0.16 | 0.036 | –1.7 |
| $\rho$ | 0.010 mm | 0.073 | 0.083 | 0.12 |
| $F_0$ | 1.0 mg/kg | 0.076 | 0.076 | 0 |

[a]$\mu_{1/2}$ and $\mu_2$ are the decay rates associated with reducing by half and doubling, respectively, the specific parameter listed. [b]To measure the effect of each parameter on the elimination rate of the fusion protein in the tumour, the ratio $E = \mu_2 - \mu_{1/2}/\mu_1$ was computed, where $\mu_1 = 0.076$ is the elimination rate when all parameters have their baseline values. The larger the deviation from zero, the more significant the parameter.

experimentally and by mathematical model simulations are comparable (Table 5). However, a significant difference was found for the amount of conjugate remaining the plasma in animals that received multiple conjugate injections. In this dosing regimen, the mathematical model predicts that 0.002% of the injected dose should be found in the plasma, but the amount found was four times higher (0.008% injected dose per gram). The reason for this apparent discrepancy is likely due to our inability to quantify exceedingly small amounts of conjugate with the enzyme assay that was utilized for conjugate quantitation. With this limitation in mind, the simulations and experimental data are in general agreement, and lead to the conclusion that the dosing strategy does not play a critical role in the amount of conjugate that is taken up within the tumour. There would be little advantage in partitioning the L49-sFv-bL dose compared to giving the conjugate in a bolus injection.

### DISCUSSION

There are many factors that can influence intratumoural localization and systemic clearance of macromolecular conjugates (Baxter and Jain, 1989, 1991). Included among them are tumour vascularity and intrastitial pressure, diffusion of the conjugate out of the vasculature, and conjugate molecular weight and blood clearance characteristics. Once the conjugate gains access to the tumour mass, distribution and retention can be affected by antigen distribution, conjugate association and dissociation rates, and processing of the conjugate once it becomes bound to the tumour cells. The net result is that therapeutic optimization studies are generally empirical and quite laborious. For example, in the mAb–enzyme/prodrug strategy, the administration of prodrug following conjugate treatment has ranged from 12 h for a fast clearing sFv fusion protein (Siemers et al, 1997), to 2 weeks for a slow clearing whole mAb conjugate (Eccles et al, 1994). It is significant that none of the published in vivo examples of this particular targeting strategy have been subject to rigorous experimental optimization. This is due to the large number of parameters involved. As a result, mathematical models that shed insight into how best in vivo experiments can be carried out are of great value.

The original model for the mAb–enzyme prodrug targeting strategy was described by Jain and co-workers (Jain et al, 1992, 1996). These investigators regarded the biological system as being comprised of three compartments, the blood pool, the tumour and the remaining tissues. A set of equations was generated from such parameters and variables as those listed in Table 1. The focus of developing this model was to delineate the main contributing factors for achieving high intratumoural drug concentrations, which were concluded to be the conjugate tumour/blood ratio and

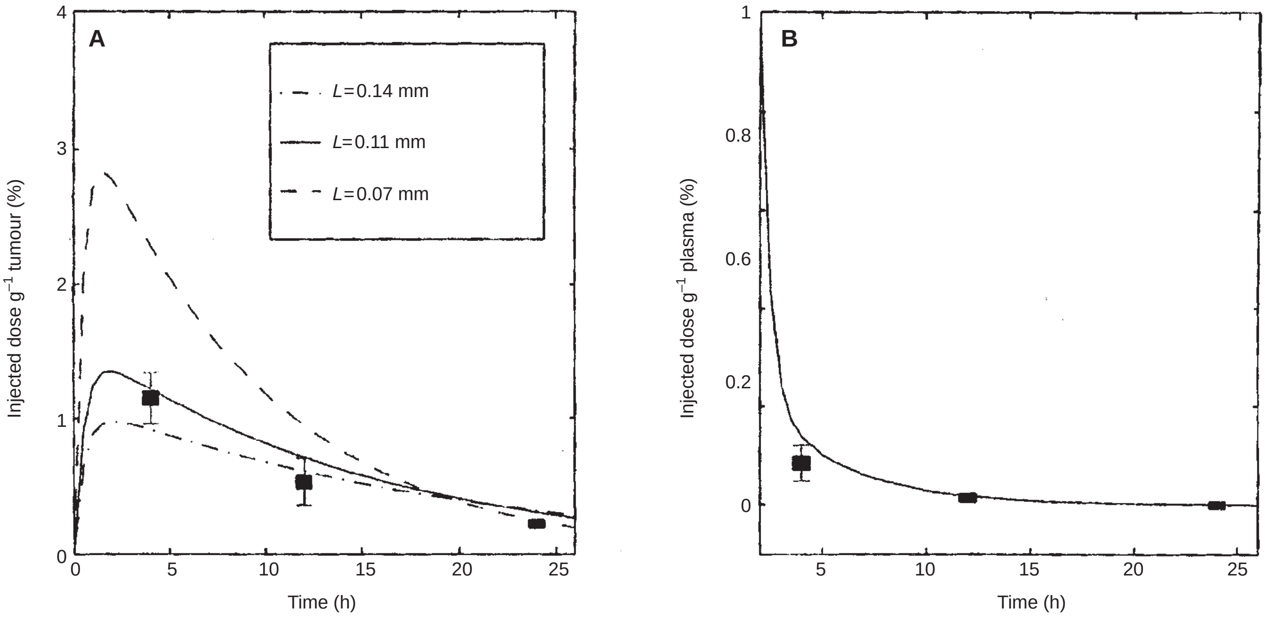

**Figure 3** Mathematical model simulation of conjugate localization in tumour tissue (**A**) and plasma (**B**) compared with the experimental findings reported by Siemers and co-workers (Siemers et al, 1997). The model data using a value of 0.11 mm for $L$ correlates with the experimentally determined data

**Table 4** Correlation of experimental localization data with the mathematical model

| | % ID $g^{-1}$ Tumour[a] | | % ID $g^{-1}$ Plasma[a] | | Ratio | |
|---|---|---|---|---|---|---|
| Time (h) | Model | Experiment | Model | Experiment | Model | Experiment |
| 4 | 1.22 | 1.15 ± 0.18 | 0.13 | 0.084 ± 0.04 | 9.38 | 15.2 ± 3.5 |
| 12 | 0.711 | 0.532 ± 0.17 | 0.017 | 0.008 ± 0.002 | 41.8 | 68.7 ± 12.7 |
| 24 | 0.308 | 0.214 ± 0.02 | 0.001 | 0.002 ± 0.000 | 308 | 111 ± 26.1 |

[a]The dose administered was 1.0 mg $kg^{-1}$ for the experiments and the model simulations.

**Table 5** Effect of dosing schedule on conjugate localization

| Administration | Schedule (Time in hours) | % ID $g^{-1}$ Tumour[a] | | % ID $g^{-1}$ Plasma[a] | |
|---|---|---|---|---|---|
| | | Model | Experiment | Model | Experiment |
| Bolus[b] | 0 | 0.31 | 0.29 ± 0.05 | 0.01 | 0.006 ± 0.002 |
| Multiple[c] | 0, 4, 8 | 0.41 | 0.32 ± 0.03 | 0.002 | 0.008 ± 0.002 |
| Continuous[d] | 0–8 | 0.41 | n.d.[e] | 0.003 | N.D.[e] |

[a]The measurements and calculations were determined 24 h post-conjugate administration. [b]A single injection of 4 mg $kg^{-1}$ was administered. [c]Three equal doses of 1.33 mg $kg^{-1}$ $injection^{-1}$ was administered. [d]Continuous infusion of 4 mg $kg^{-1}$ of conjugate over an 8 h period was simulated. [e]ND, Not determined.

the enzyme kinetic parameters, $K_m$ and $V_{max}$. We wished to expand upon this model and to focus specifically on the parameters that affected intratumoural conjugate uptake, retention and localization index. A secondary aspect of the work was to use the model to predict the effects of various conjugate dosing strategies.

Of all the parameters considered, tumour vasculature, vessel permeability and the conjugate dissociation ratio, $\frac{k_d}{k_a A_0}$, were most important in determining conjugate localization. The model we describe predicts that increases in the inter-capillary half-distance, $L$, will decrease the concentration of conjugate in the tumour as a power law. Retention of conjugate in the tumour mass is most significantly influenced by the rate of association and dissociation from cell-surface antigens. Reducing the conjugate dissociation ratio by 50% will double the half-life in the tumour. The model therefore predicts that the highest intratumoural conjugate localization will involve high affinity mAbs that target well-vascularized tumours.

The model was validated experimentally and an excellent correlation was found to exist between the model predictions and the experimental data (Figure 3). The comparison made in Figure 3 does not represent a least-squares parameter-optimizing curve fit to the localization data, but rather represents simulations using fixed parameters estimated from other experiments. The model enabled us to make predictions concerning the differences that would be expected if the conjugate was administered in a single bolus injection, in partitioned doses, or as a continuous infusion. The model predicted that the three dosing regimens would yield roughly the same intratumoural conjugate concentrations. This was confirmed experimentally for both bolus and multiple injection strategies (Table 5).

The mathematical approach and the general conclusions from this work can be applied to other macromolecules that bind to receptors in solid tumour masses. The critical information needed to use the model include knowledge of the plasma clearance rates, the binding constants, and the average inter-capillary half-distance. Using this model, it is possible to predict the total amount of conjugate that will bind and the amount within a tumour at a given time post-injection. This information will prove useful in designing optimal dosing strategies for targeted drug therapy. We are now extending the model to include predictions of intratumoural drug concentrations that result after the administration of prodrug.

### ACKNOWLEDGEMENTS

This work has been supported in part by NSF grants DMS-9306108 (SRL) and DMS-9500766 (SRL, JDM), and by an NSF Graduate Fellowship (TLJ).

### REFERENCES

Bagshawe KD and Begent RHJ (1996) First clinical experience with ADEPT. *Adv Drug Delivery Rev* **22**: 365–367

Baxter LT and Jain RK (1989) Transport of fluid and macromolecules in tumors. I. role of interstitial pressure and convection. *Microvasc Res* **37**: 77–104

Baxter LT and Jain RK (1991a) Transport of fluid and macromolecules in tumors. III. Role of binding and metabolism. *Microvasc Res* **41**: 5–23

Baxter LT and Jain RK (1991b) Transport of fluid and macromolecules in tumors. IV. a microscopic model of the perivascular distribution. *Microvasc Res* **41**: 252–272

Baxter LT and Jain RK (1996) Pharmacokinetics analysis of the microscopic distribution of enzyme-conjugated antibodies and prodrugs: comparison with experimental data. *Br J Cancer* **73**: 447–456

Baxter LT, Yuan F and Jain RK (1992) Pharmacokinetic analysis of the perivascular distribution of bifunctional antibodies and haptens: comparison with experimental data. *Cancer Res* **52**: 5838–5844

Bosslet K, Czech J and Hoffmann D (1994) Tumor-selective prodrug activation by fusion protein-mediated catalysis. *Cancer Res* **54**: 2151–2159

Eccles SA, Court WJ, Box GA, Dean CJ, Melton GR and Springer CJ (1994) Regression of established breast carcinoma xenografts with antibody-directed enzyme prodrug therapy against c-erb-b2 p185. *Cancer Res* **54**: 5171–5177

Gerlowski LE and Jain RK (1986) Microvascular permeability of normal and neoplastic tissues. *Microvas Res* **31**: 288–305

Kerr DE, Schreiber GJ, Vrudhula BM, Svensson HP, Hellstrom KE and Senter PD (1995) Regressions and cures of melanoma xenografts following treatment with monoclonal antibody β-lactamase conjugates in combination with anticancer prodrugs. *Cancer Res* **55**: 3558–3563

© 1999 Cancer Research Campaign

Krogh A (1919) The number and distribution of capillaries in muscles with calculations of the oxygen pressure head necessary for supplying the tissue. *J of Physiol* **52**: 409–415

Melton RG and Sherwood RF (1996) Antibody-enzyme conjugates for cancer therapy. *J Natl Cancer Inst* **88**: 153–165

Meyer DL, Jungheim LN, Law KL, Mikolajczyk SD, Shepherd TA, Mackensen DG, Briggs SL and Starling JJ (1992) Site-specific prodrug activation by antibody-β-lactamase conjugates: regression and long term growth inhibition of human colon carcinoma xenograft models. *Cancer Res* **53**: 3956–3963

Niculescu-Duvaz I and Springer CJ (1996) Antibody-directed enzyme prodrug therapy (ADEPT): a targeting strategy in cancer chemotherapy. *Curr Med Chem* **2**: 687–706

Nugent LJ and Jain RK (1984) Extravascular diffusion in normal and neoplastic tissues. *Cancer Res* **44**: 238–244

O'Callaghan DE, Morris A, Kirby S and Shingler AH (1972) Novel method for detection of β-lactamases by using a chromogenic cephalosporin substrate. *Antimicrob Agents Chemother* **1**: 283–288

Pietersz GA, Rowland A, Smyth MJ and McKenzie IF (1994) Chemoimmuno-conjugates for the treatment of cancer. *Adv Immunol* **56**: 301–387

Senter PD and Svensson HP (1997) A summary of monoclonal antibody–enzyme/prodrug combinations. *Adv Drug Delivery Rev* **22**: 341–349

Senter PD, Wallace PM, Svensson HP, Vrudhula VM, Kerr DE, Hellstrom I and Hellstrom KE (1993) Generation of cytotoxic agents by targeted enzymes. *Bioconjugate Chem* **4**: 3–9

Siemers NO, Kerr DE, Yarnold S, Stebbins M, Vrudhula VM, Hellstrom I, Hellstrom KE and Senter PD (1997) L49-sfv-β-lactamase, a single chain anti-p97 antibody fusion protein. *Bioconjugate Chem* **8**: 510–519

Springer CJ, Bagshawe KD, Sharma SK, Searle F, Boden JA, Antoniw P, Burke PJ, Rogers GT, Sherwood RF and Melton RG (1991) Abalation of human choriocarcinoma xenografts in nude mice by antibody-directed enzyme prodrug therapy (ADEPT) with three novel compounds. *Eur J Cancer* **27**: 1361–1366

Svensson HP, Vrudhula VM, Emswiler JE, MacMaster JF, Cosand WL, Wallace PM and Senter PD (1995) In vitro and in vivo activities of doxorubicin prodrug in combination with monoclonal antibody β-lactamase conjugates. *Cancer Res* **55**: 2357–2365

Wallace PM, MacMaster JF, Smith V, Kerr DE, Senter PD and Cosand WL (1994) Intratumoral generation of 5-fluorouracil mediated by an antibody-cytosine deaminase conjugate in combination with 5-fluorocytosine. *Cancer Res* **54**: 2719–2723

*© 1999 Cancer Research Campaign*
